# Whole-genome SNP association analysis of reproduction traits in the Finnish Landrace pig breed

**DOI:** 10.1186/1297-9686-43-42

**Published:** 2011-12-01

**Authors:** Pekka Uimari, Anu Sironen, Marja-Liisa Sevón-Aimonen

**Affiliations:** 1 Agrifood Research Finland, MTT, Biotechnology and Food Research, FI-36100 Jokioinen, Finland

## Abstract

**Background:**

Good genetic progress for pig reproduction traits has been achieved using a quantitative genetics-based multi-trait BLUP evaluation system. At present, whole-genome single nucleotide polymorphisms (SNP) panels provide a new tool for pig selection. The purpose of this study was to identify SNP associated with reproduction traits in the Finnish Landrace pig breed using the Illumina PorcineSNP60 BeadChip.

**Methods:**

Association of each SNP with different traits was tested with a weighted linear model, using SNP genotype as a covariate and animal as a random variable. Deregressed estimated breeding values of the progeny tested boars were used as the dependent variable and weights were based on their reliabilities. Statistical significance of the associations was based on Bonferroni-corrected *P*-values.

**Results:**

Deregressed estimated breeding values were available for 328 genotyped boars. Of the 62 163 SNP in the chip, 57 868 SNP had a call rate > 0.9 and 7 632 SNP were monomorphic. Statistically significant results (*P*-value < 2.0E-06) were obtained for total number of piglets born in first and later parities and piglet mortality between birth and weaning in later parity, and suggestive associations (*P*-value < 4.0E-06) for piglet mortality between birth and weaning in first parity, number of stillborn piglets in later parity, first farrowing interval and second farrowing interval. Two of the statistically significant regions for total number of piglets born in first and later parities are located on chromosome 9 around 95 and 79 Mb. The estimated SNP effect in these regions was approximately one piglet between the two homozygote classes. By combining the two most significant SNP in these regions, favourable double homozygote animals are expected to have 1.3 piglets (*P*-value = 1.69E-08) more than unfavourable double homozygote animals. A region on chromosome 9 (66 Mb) was statistically significant for piglet mortality between birth and weaning in later parity (0.44 piglets between homozygotes, *P*-value = 6.94E-08).

**Conclusions:**

Three separate regions on chromosome 9 gave significant results for litter size and pig mortality. The frequencies of favourable alleles of the significant SNP are moderate in the Finnish Landrace population and these SNP are thus valuable candidates for possible marker-assisted selection.

## Background

Estimated breeding values (EBV) based on best linear unbiased prediction (BLUP) for total numbers of piglets born and farrowing intervals have been available to Finnish pig breeders since 1991 [1]. The current multi-trait BLUP evaluation and fertility index also includes pig mortality and age at first farrowing [2]. During the last decade, a favourable genetic trend has been observed in the Finnish Landrace pig population for total litter size in terms of number of piglets weaned per litter (0.1 piglet) [3]. Selection based on quantitative genetic theory and the BLUP method has been successful in improving female reproduction traits. However, genetic improvement of reproduction traits, which have a low heritability and sex-limited expression, constitutes a real challenge for animal breeders and requires a better understanding of the genetic architecture of these traits to allow selection on genetic variants affecting these traits [4].

Over the past twenty years, several microsatellite-based linkage studies have been conducted to locate quantitative trait loci (QTL) that affect pig reproduction traits. The results are listed in the Pig Quantitative Trait Locus database (Pig QTLdb, http://www.genome.iastate.edu/cgi-bin/QTLdb/SS/index) [5,6]. Through a collaborative effort between the International Porcine SNP Chip Consortium and Illumina (CA, San Diego), researchers in pig breeding have now access to a whole-genome SNP panel, which makes it possible to study in greater detail the genetic architecture of reproduction traits in pigs [7].

The objective of this study was to identify SNP associated with female reproduction traits in the Finnish Landrace pig breed. Significant SNP can then be incorporated into the national evaluation and selection scheme. The analysis is based on 328 progeny-tested artificial insemination (AI) boars genotyped with the Illumina PorcineSNP60 BeadChip.

## Methods

### Animal material

The study included 328 Finnish Landrace AI boars born between 1996 and 2009. The average and maximum numbers of daughters per sire were 141 and 782, respectively. All boars were related to each other. The data included 114 sires with genotyped sons, for which the average and maximum numbers of sons per sire were 2.2 and 10, respectively.

### DNA extraction and genotyping

DNA was extracted either from hair follicles or from semen using a DNeasy Blood & Tissue kit (Qiagen, Helsinki, Finland). For more information on extraction methods see Sironen et al. [8]. Expected DNA concentrations were 100 ng/μL for semen and 50 ng/μL for hair follicles. For each sample, 20 μL of DNA sample was sent out for genotyping at the FIMM (Institute for Molecular Medicine Finland, Helsinki, Finland) using the PorcineSNP60 BeadChip.

### Phenotypes and statistical method

Nine female reproductive traits were studied: total number of piglets born in first (TNB1) and later parities (TNB2), number of stillborn piglets in first (NSB1) and later parities (NSB2), piglet mortality between birth and weaning in first (PM1) and later parities (PM2), age at first farrowing (AFF), first farrowing interval (FFI), and second farrowing interval (SFI). EBV for all nine traits were obtained for each genotyped AI boar from the national breeding value evaluation (multi-trait BLUP). The linear model for TNB1, TNB2, NSB1, NSB2, PM1, and PM2 used in the national evaluation includes herd-year, year-month, type of insemination, litter breed, and age at farrowing as fixed effects, and litter sire, permanent environmental effects, and additive genetic (animal) effects as random effects. Additionally, parity number was included as a fixed effect for TNB2, NSB2, and PM2. The linear model for AFF, FFI, and SFI included herd-year and herd-month as fixed effects and animal as a random effect. The models for FFI and SFI also included the effect of dam breed.

Prior to SNP association analysis, unstandardized EBV were deregressed and corresponding weights were calculated based on individual and parental EBV and reliabilities [9]. The proportion of genetic variance not explained by markers, due to partial marker coverage of the genome and incomplete linkage disequilibrium between markers and causal genes, was fixed at 0.5 (parameter c in Garrick et al. [9]).

The association of SNP with deregressed EBV was studied using a mixed linear model, for each SNP separately. The model included a fixed SNP effect and a random polygenic effect to account for residual genetic variance not explained by the SNP in the model and the relationship between animals in the data. The model used was:

$${\text{y}}_{\text{i}}=\mu +\text{b}\times {\text{x}}_{\text{i}}+{\text{a}}_{\text{i}}+{\text{e}}_{\text{i}},$$

where y_i _is the deregressed EBV; x_i _is the number of minor alleles (0, 1, or 2); b is the corresponding regression coefficient; a_i _is a random polygenic effect with covariance structure a_i _~N(0, **A**σ^2^_a_), where **A **is the additive relationship matrix and σ^2^_a _is the polygenic variance; and e_i _is a random residual effect with e_i _~N(0, **I**σ^2^_e_/w_i_), where **I **is an identity matrix, σ^2^_e_ is the residual variance, and w_i _is the weight. The analyses were performed using the AI-REML method in the DMU program package [10]. Variance components were estimated separately for each SNP. The estimated heritabilities based on deregressed EBV were generally smaller than the ones used in national breeding value estimation (e.g. h^2 ^= 0.07 vs. 0.10 for TNB1) but were quite constant across SNP.

Statistical significance of the associations was based on Bonferroni-corrected *P*-values. This method treats individual tests as independent and thus is very conservative for data for which the correlation (linkage disequilibrium) between tests (SNP) is high; the linkage disequilibrium (r^2^) between adjacent SNP in the Finnish Landrace population is 0.43 [11]. Aiming for an overall false positive rate of 0.05 and considering 50 000 to 25 000 independent tests, the point-wise *P*-value should be between 1.0E-06 and 2.0E-06. In this article, individual SNP with a *P*-value of 2.0E-06 or less were considered statistically significant, and SNP with a *P*-value of 4.0E-06 or less as suggestive. More precise estimates of multiple-test-corrected *P*-values can be obtained by a permutation procedure but this was not possible for this research because of its high computation demand.

## Results

### SNP quality

A total of 390 animals were originally genotyped; 366 animals had a call rate above the commonly used limit of 90% and five samples (DNA extracted from hair follicles) had a call rate of 0. Only the samples with a call rate equal or above to 0.90 and with available national EBV were used in the association analysis (328 animals).

The Illumina PorcineSNP60 BeadChip contains 62 163 SNP http://www.illumina.com. One quarter of these had a call rate of 1.0, and 57 868 SNP had a call rate equal to or above 0.9 and were used in the association analyses. A large proportion of the SNP (7 632) were monomorphic (for these, no estimate for SNP effect is available) and 7 642 SNP had a minor allele frequency below 0.05. Most of the SNP with a low frequency were evenly distributed across the genome, but regions with low polymorphism longer than 1 Mb were also detected on different chromosomes. These low-polymorphism regions could be the result of selection, random genetic drift, or a bottleneck effect. Otherwise, the distribution of minor allele frequency was uniform across SNP. The SNP were mapped to pig genome build 9 (Sscrofa9, http://www.ensembl.org).

### Association results

**Figure 1:**
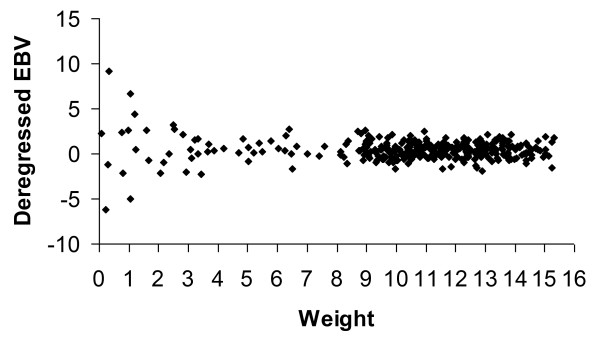
**Distribution of deregressed EBV and weights for total number of piglets born (TNB1)**.

For each trait, animals with a weight of the deregressed EBV less than 1.0 were removed from the analysis. The choice of this limit was arbitrary and subsequent analyses showed that inclusion or exclusion of these animals in the data had no real effect on the association results because of their large residual variance (small weight) in the linear mixed model equations. Figure 1 describes the distribution of deregressed EBV and weights for TNB1, and Table 1 gives the number of observations, mean, and standard deviation of the studied traits. Also the mean reliabilities of the original EBV are presented in Table 1.

**Table 1:** Descriptive statistics of the analyzed deregressed EBV

**Trait and unit**^**1**^	N	Mean	Standard deviation	Min - Max	Mean/mode of **reliability**^**2**^
TNB1, piglet	319	0.43	1.03	-4.97 - 6.61	0.81/0.92
NSB1, piglet	320	0.07	0.48	-2.16 - 2.62	0.77/0.86
PM1, piglet	313	0.04	0.52	-3.06 - 1.63	0.72/0.79
TNB2, piglet	315	0.36	0.98	-2.97 - 3.73	0.83/0.93
NSB2, piglet	311	0.05	0.50	-1.98 - 4.16	0.80/0.78
PM2, piglet	306	0.03	0.57	-2.11 - 2.93	0.74/0.86
AFF, day	307	16.79	11.76	-21.12 - 61.96	0.84/0.89
FFI, day	300	5.01	7.10	-19.92 - 35.27	0.70/0.70
SFI, day	288	2.21	7.07	-22.73 - 48.91	0.61/0.60

^1^TNB1 = total number of piglets born in first parity; NSB1 = number of stillborn piglets in first parity; PM1 = piglet mortality between birth and weaning in first parity; TNB2 = total number of piglets born in later parities; NSB2 = number of stillborn piglets in later parities; PM2 = piglet mortality between birth and weaning in later parities; AFF = age at first farrowing; FFI = first farrowing interval; SFI = second farrowing interval; observations with weight < 1 were discarded

^2^mean and mode of reliabilities of the original EBV

**Figure 2:**
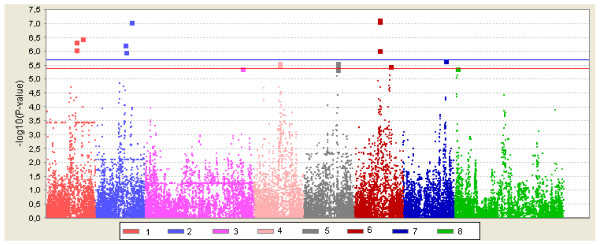
***P*-values (-log10(P-value)) for SNP effects on chromosomes with significant or suggestive SNP**. The threshold value for significant association is indicated by the blue horizontal line and that for suggestive association by the red horizontal line. 1: SSC9 and total number of piglets born in first parity (TNB1); 2: SSC9 and total number of piglets born in later parities (TNB2); 3: SSC1 and number of stillborn piglets in later parities (NSB2); 4: SSC9 and piglet mortality between birth and weaning in first parity (PM1); 5: SSC7 and piglet mortality between birth and weaning in later parities (PM2); 6: SSC9 and PM2; 7: SSC4 and first farrowing interval (FFI); 8: SSC1 and second farrowing interval (SFI).

**Figure 3:**
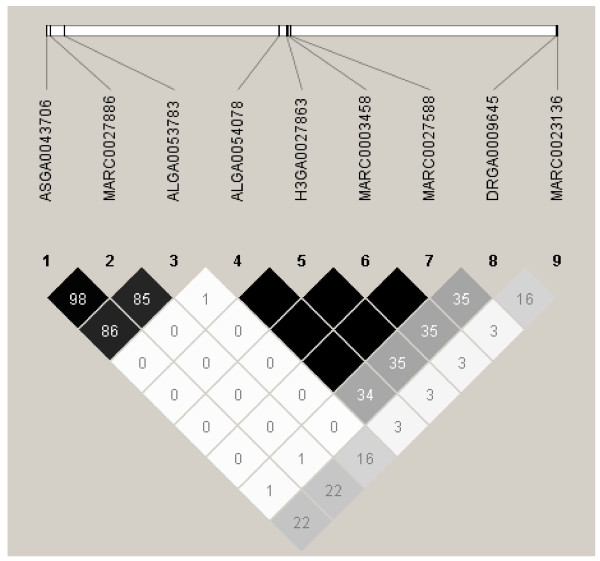
**Haploview plot of linkage disequilibrium (r^2^) between significant and suggestive SNP on chromosome 9**. A black diamond without a number represents complete linkage disequilibrium between SNP (r^2 ^= 1).

Significant associations were observed for TNB1, TNB2, and PM2, and suggestive associations for PM1, NSB2, FFI, and SFI (Table 2 and Figure 2). Two chromosomal regions on chromosome 9 (around 79 Mb and 95 Mb, based on Sscrofa9) were statistically significant for litter size of first and later parities (TNB1 and TNB2). The estimated effect of SNP in these two regions of chromosome 9 was approximately 1 piglet between the two homozygote classes (e.g. SNP DRGA0009645; AA-genotypes vs. GG-genotypes). SNP ALGA0054078, H3GA0027863, MARC0003458, and MARC0027588 (79 Mb) were in complete linkage disequilibrium with each other and in moderate linkage disequilibrium (r^2 ^= 0.36) with DRGA0009645 (95 Mb) (Figure 3). During the last 15 years, the frequency of favourable alleles increased from 0.10 to 0.19 for SNP in the 79 Mb region and from 0.14 to 0.22 for DRGA0009645 (Table 2). This positive trend in allele frequencies is in good agreement with the overall increase in litter size observed in the Finnish Landrace breed over the same period.

**Table 2:** Allele effects and *P*-values of significant (in bold face) and suggestive SNP

**Trait**^**1**^	Marker	**CHR**^**2**^	Position (bp)	**CR**^**2**^	**MAF1**^**2**^	**MAF2**^**2**^	**MAF3**^**2**^	N	**b**^**3**^	**S.E**.	P-value
TNB1	ALGA0054078	9	79167181	0.99	0.10	0.13	0.19	316	0.55	0.11	**4.24E-07**
TNB1	H3GA0027863	9	79597323	1.00	0.10	0.13	0.19	319	0.52	0.10	**8.21E-07**
TNB1	MARC0003458	9	79667545	1.00	0.10	0.13	0.19	319	0.52	0.10	**8.21E-07**
TNB1	MARC0027588	9	79869109	1.00	0.10	0.13	0.19	319	0.52	0.10	**8.21E-07**
TNB1	DRGA0009645	9	95379632	1.00	0.14	0.13	0.22	319	0.51	0.10	**3.21E-07**

TNB2	ALGA0054078	9	79167181	0.99	0.10	0.13	0.19	312	0.55	0.11	**5.62E-07**
TNB2	H3GA0027863	9	79597323	1.00	0.10	0.13	0.19	315	0.53	0.11	**1.03E-06**
TNB2	MARC0003458	9	79667545	1.00	0.10	0.13	0.19	315	0.53	0.11	**1.03E-06**
TNB2	MARC0027588	9	79869109	1.00	0.10	0.13	0.19	315	0.53	0.11	**1.03E-06**
TNB2	DRGA0009645	9	95379632	1.00	0.14	0.13	0.22	315	0.55	0.10	**8.45E-08**

NSB2	ALGA0009013	1	261069838	1.00	0.36	0.40	0.54	310	-0.15	0.03	3.97E-06
NSB2	ASGA0006533	1	261117360	1.00	0.36	0.40	0.54	310	-0.15	0.03	3.97E-06

PM1	ASGA0043706	9	65533618	0.94	0.34	0.39	0.39	295	-0.18	0.04	2.92E-06
PM1	MARC0027886	9	65764105	1.00	0.37	0.40	0.40	313	-0.18	0.04	2.50E-06

PM2	MARC0016206	7	89927000	1.00	0.21	0.22	0.15	306	-0.21	0.04	2.89E-06
PM2	ALGA0042932	7	90018155	1.00	0.20	0.21	0.15	306	-0.22	0.04	2.51E-06
PM2	ASGA0043706	9	65533618	0.94	0.34	0.39	0.39	288	-0.22	0.04	**6.94E-08**
PM2	MARC0027886	9	65764105	1.00	0.37	0.40	0.40	306	-0.21	0.04	**7.98E-08**
PM2	ALGA0053783	9	66630460	0.99	0.33	0.36	0.38	303	-0.20	0.04	**8.85E-07**
PM2	MARC0023136	9	95402048	0.99	0.35	0.47	0.52	304	-0.18	0.04	3.30E-06

FFI	H3GA0014078	4	112914704	1.00	0.57	0.37	0.23	300	2.40	0.49	2.01E-06

SFI	ALGA0000673	1	8282135	1.00	0.24	0.20	0.23	288	2.38	0.50	3.83E-06

^1^TNB1 = total number of piglets born in first parity; TNB2 = total number of piglets born in later parities; NSB2 = number of stillborn piglets in later parities; PM1 = piglet mortality between birth and weaning in first parity; PM2 = piglet mortality between birth and weaning in later parities; FFI = first farrowing interval; SFI = second farrowing interval

^2^CHR = chromosome; CR = call rate; MAF1 = minor allele frequency of animals born in 1996 - 1999; MAF2 = minor allele frequency of animals born in 2000 - 2004; MAF3 = minor allele frequency of animals born after 2004

^3^the regression coefficient is expressed as a dose effect of the minor allele

**Figure 4:**
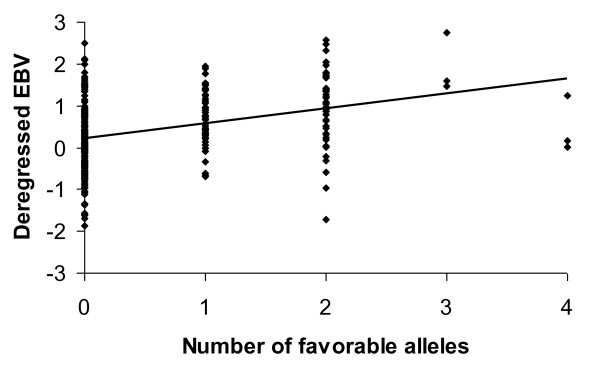
**Effect of significant SNP on TNB1**. Distribution of deregressed EBV for total number of piglets born (TNB1) against the number of favourable alleles for SNP H3GA0027863 and DRGA0009645; deregressed EBV with weight less than 5.0 were discarded.

When the two regions were combined in the analysis and the regression variable corresponded to the number of favourable alleles (A) in H3GA0027863 and DRGA0009645 (possible values 0, 1, 2, 3, and 4), the estimated SNP effect was 0.32, with a *P-*value of 1.69E-08. Thus, animals that are double homozygotes AA for H3GA0027863 and DRGA0009645 are expected to have 1.28 (4*0.32) more piglets than animals that are double homozygotes GG. To test the sensitivity of the analysis, observations with a weight less than 5 were discarded. For this analysis, the estimated SNP effect was also 0.32, with a *P-*value of 1.08E-08. Figure 4 shows the distribution of the deregressed EBV of TNB1 for animals with different combinations of genotypes for H3GA0027863 and DRGA0009645 (deregressed EBV with a weight less than 5.0 were discarded).

Another region on chromosome 9 (around 66 Mb, based on Sscrofa9) was statistically significant for piglet mortality before weaning (PM1 and PM2, Table 2). SNP in this region were in strong linkage disequilibrium with each other (r^2 ^ranging from 0.85 to 0.94) but nearly in linkage equilibrium with the SNP in region 79 Mb on the same chromosome that were significant for litter size (r^2 ^< 0.01) (Figure 3). Moreover, the SNP that gave suggestive evidence for PM2 at 95.4 Mb on chromosome 9 (MARC0023136) was in weak linkage disequilibrium (r^2 ^= 0.17) with a SNP in the same proximal area (DRGA0009645) that was significant for litter size (Figure 3). *P*-values for the association of MARC0023136 on TNB1 and TNB2 were 0.002 and 0.0005, respectively, reflecting the observed linkage disequilibrium between MARC0023136 and DRGA0009645. The low linkage disequilibrium between adjacent DRGA0009645 and MARC0023136, which were located 22 kb from each other, may be due to mapping errors in build 9 (Sscrofa9, http://www.ensembl.org). The frequencies of favourable alleles for pig mortality have increased during the last 15 years. This could be due either to random drift or, given the fact that the SNP are associated with the trait under selection, to selection force (Table 2).

Suggestive evidence for associations between SNP and female reproduction traits was also observed for number of stillborn piglets in later parities (NSB2) on chromosome 1, for pig mortality in later parities (PM2) on chromosome 7, for first farrowing interval (FFI) on chromosome 4, and for later farrowing interval (SFI) on chromosome 1 (Table 2).

## Discussion

Good genetic progress for pig reproduction traits has been achieved using a quantitative genetic-based multi-trait BLUP evaluation system. Marker-assisted selection [12] was expected to boost selection efficiency to a new level [4]. As is well known, these promises have not been fulfilled, mainly due to the lack of markers strongly linked to QTL. High-density SNP genotyping technology provides a new tool to select elite animals for breeding. SNP are also more powerful to study the genetic background of traits than microsatellites because they have a better genomic coverage and can provide information on historical linkage disequilibrium with potential QTL, whereas microsatellite linkage studies were based on more recent linkage within families. High-density SNP genotypes are primarily incorporated into breeding programs through the use of genomic selection instead of traditional marker-assisted selection, particularly in dairy cattle breeding [13].

Marker-assisted selection on known marker-QTL associations and genomic selection on an overall sum of marker effects across the genome both rely on strong linkage disequilibrium between markers and QTL. Finnish pig breeds are very suitable for SNP association studies because of their high linkage disequilibrium over the typical distances between SNP in the PorcineSNP60 BeadChip [11] and the homogeneity of the population. Other important factors affecting the power of association studies, beyond the actual genetic architecture of the trait, are the number of genotyped animals and the reliability of the observations used in statistical analysis. In this study, over 300 AI boars with an average number of 141 daughters per sire were available for association analyses. Given that the number of genotyped animals is the same in both scenarios, for traits with a low heritability, a half-sib design, as used here is more powerful for association studies than a direct design, in which the same animals are both genotyped and phenotyped [14]. Given the number of genotyped boars and daughters per sire, a direct design would have required several hundreds to several thousands more genotyped animals to achieve the same power as the half-sib design used here (e.g. with an average number of 40 daughters per sire, h^2 ^= 0.1, a QTL that explains 5% of the phenotypic variation, and a significance value of 2.0E-06, approximately 650 genotyped sows are needed to achieve the same power (0.8) as 130 genotyped AI boars [14]). Although a larger number of genotyped sires would have improved the power, the results show that the data size was sufficient to discover three new regions on chromosome 9 with effects on litter size and piglet mortality with very small *P*-values. The reported effects of these SNP are most likely overestimated due to the "winner's curse" effect [15] that is commonly observed in initial genome-wide association studies. A larger population study is still needed to precisely estimate the effects and gene actions of these chromosomal regions on litter size and pig mortality.

Annotation of the SNP was based on the pig genome build 9 (Sscrofa9, http://www.ensembl.org). It is well known that build 9 contains errors in the actual position of the SNP and even in the order of the SNP, as suggested by the linkage disequilibrium pattern of the significant SNP on chromosome 9 in this study. However, single SNP association tests are not sensitive to mapping errors. Thus, results (*P*-values) presented here hold even with an updated pig genome build but the candidate genes that are in the proximity of the significant SNP may change from the ones presented here.

The three regions on chromosome 9 (around 65, 79 and 95 Mb) that were significant for female reproduction traits in this study are within the same region as that reported for ovulation rate by Rohrer et al. [16] (57 to 122 cM, with a peak at 67 cM), based on their whole-genome microsatellite study of Chinese Meishan × European White composite line crossbreds. A significant region on chromosome 9 was also reported for ovulation rate in another microsatellite-based genome scan of an F2 cross of two selected experimental lines [17]. However, this 1 cM region does not overlap with the region reported here or by Rohrer et al. [16]. Yet another microsatellite genome scan of Meishan × Large White F2 population revealed two significant regions on chromosome 9 for female reproduction traits: a region at 127 cM was significant for ovulation rate and one at 36 cM for number of viable embryos and embryo survival [18]. Neither of these regions overlaps with the significant regions in this study.

The suggestive region for NSB2 at the end of chromosome 1 that was found in this study has been reported to carry a QTL for age at puberty [18] but not for number of stillborn piglets. The *estrogen receptor 1 *(*ESR1*) gene on chromosome 1 (15186680 to 15450209 bp) is a good candidate gene for all reproduction traits. However, none of the SNP in this region gave significant or suggestive *P*-values for any of the studied traits here. The best SNP (ALGA0000673 for SFI) is located 7 Mb from the *ESR1 *gene. A QTL for number of stillborn has been reported on chromosome 7 [19] and a QTL for ovulation rate on chromosome 4 [18].

It is interesting that the genome regions that reached statistical significance in our study are all on chromosome 9. Without further studies, it is difficult to say whether this is by chance or whether there is some biological or population-based mechanism that explains this result. No linkage disequilibrium was observed between the significant regions on chromosome 9 at the population level, which implies that the regions have segregated independently in the Finnish Landrace population and thus rules out the hitchhiking effect as the most likely explanation.

The region on chromosome 9 that is associated with TNB1 and TNB2 contains several potential candidate genes that may contribute to the physiology of variation in sow fertility. The *aryl hydrocarbon receptor *(*AhR*) gene between positions 81024216 and 81081616 bp is involved in folliculogenesis, gonadotrophin receptor expression, proliferation of granulosa cells, and intraovarian estrogen signalling [20]. Its well-balanced activity is necessary for normal ovarian function. Additionally, it has been found that *AhR *knockout mice have reduced fertility due to disturbed follicle development, with significantly fewer pre-antral and antral follicles and less ovulations compared with wild-type mice [21]. Another candidate gene for litter size is *interleukin 6 *(*IL6*), which is located on chromosome 9 between positions 85801970 and 85806347 bp. IL6 is a multifunctional cytokine that regulates various aspects of the immune response and is also expressed during the ovulation process [22]. IL6 has been shown to serve as a potent regulator of ovarian cumulus cell function and cumulus cell oocyte complex expansion, and it may mediate some of its effects [23]. Furthermore, the *protein tyrosine phosphatase non-receptor type 12 *(*PTPN12, PTP-PEST*) gene between positions 95427072 and 95467282 bp has been shown to play an essential role in early murine embryogenesis. PTPN12 functions in embryonic vascularization, mesenchyme formation, neurogenesis, and early liver development [24], and may thus affect embryo survival. However, identification of the causal genes for litter size and piglet mortality traits in the reported regions on chromosome 9 requires additional work.

## Conclusions

To conclude, this whole-genome SNP association study using 328 Finnish Landrace AI boars revealed three highly significant regions on chromosome 9 with effects on litter size and piglet mortality. Suggestive *P*-values were also observed on chromosomes 1, 4, and 7 for second and first farrowing intervals and for piglet mortality in later parities, respectively. The frequencies of favourable alleles of the significant SNP are still moderate in the Finnish Landrace population. Thus, if these initial findings are confirmed, the specified SNP will be valuable in the national breeding program through their use in marker-assisted selection.

## Competing interests

The authors declare that they have no competing interests.

## Authors' contributions

PU undertook the statistical analysis and drafted the paper. AS participated in the discussion of the results and genotyping the animals. MLSA prepared the data and participated in the statistical analysis. All authors read and approved the final manuscript.
